# Probing the Nature of Single-Photon Emitters in a WSe_2_ Monolayer by Magneto-Photoluminescence Spectroscopy

**DOI:** 10.1021/acs.nanolett.4c03686

**Published:** 2024-10-10

**Authors:** Caique Serati de Brito, Bárbara L. T. Rosa, Andrey Chaves, Camila Cavalini, César R. Rabahi, Douglas F. Franco, Marcelo Nalin, Ingrid D. Barcelos, Stephan Reitzenstein, Yara Galvão Gobato

**Affiliations:** † Department of Physics, Federal University of São Carlos, São Carlos, SP 13565-905, Brazil; ‡ Institute of Solid State Physics, Technische Universität Berlin, 10623 Berlin, Germany; ¶ Departamento de Física, Universidade Federal do Ceará, 60455-760 Fortaleza, Ceará, Brazil; § Department of Physics & NANOlab Center of Excellence, University of Antwerp, Groenenborgerlaan 171, B-2020, Antwerp, Belgium; ∥ Institute of Chemistry, São Paulo State University—UNESP, CEP 14800-060 Araraquara, SP, Brazil; ⊥ Brazilian Synchrotron Light Laboratory (LNLS), Brazilian Center for Research in Energy and Materials, Campinas (CNPEM), 13083-100 Campinas, SP, Brazil

**Keywords:** Two-Dimensional Materials, Transition Metal Dichalcogenides, Strain Engineering, Single-Photon Emitters, Magneto-Optics

## Abstract

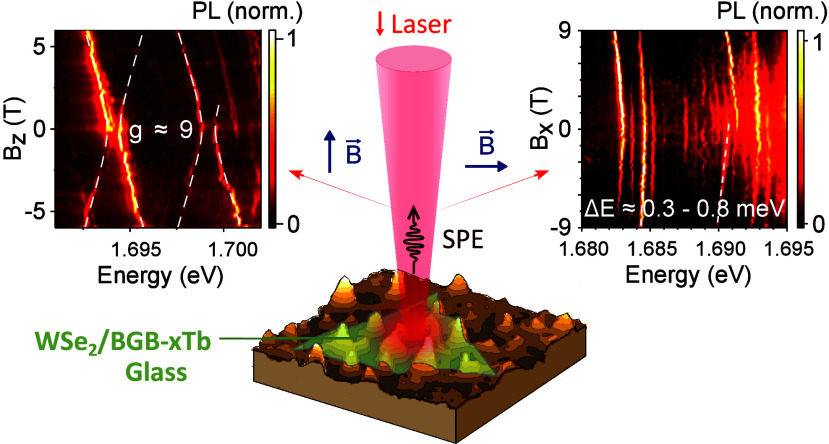

Monolayer transition metal dichalcogenides (TMDs) have emerged as promising materials to generate single-photon emitters (SPEs). While there are several previous reports in the literature about TMD-based SPEs, the precise nature of the excitonic states involved in them is still under debate. Here, we use magneto-optical techniques under in-plane and out-of-plane magnetic fields to investigate the nature of SPEs in WSe_2_ monolayers on glass substrates under different strain profiles. Our results reveal important changes on the exciton localization and, consequently, on the optical properties of SPEs. Remarkably, we observe an anomalous PL energy redshift with no significant changes of photoluminescence (PL) intensity under an in-plane magnetic field. We present a model to explain this redshift based on intervalley defect excitons under a parallel magnetic field. Overall, our results offer important insights into the nature of SPEs in TMDs, which are valuable for future applications in quantum technologies.

Two-dimensional (2D) transition metal dichalcogenides (TMDs) are a fascinating class of materials with unique physical properties, possessing potential applications in optoelectronics, spintronics, and quantum technology.^1−8^ Recently, there has been increasing interest in using 2D materials as solid-state sources of single-photon emitters (SPEs), because of their advanced properties and easy integration with photonic systems.^9−23^

Despite several experimental reports of SPEs in WSe_2_ monolayers,^9−12,16,17,21,24−26^ the fundamental mechanisms driving this phenomenon are still under investigation. Usually, two main requirements are suggested for the observation of SPEs in WSe_2_: (i) the presence of local strain and (ii) a significant density of defects such as Se vacancies.^22,27,28^ The presence of strain localizes dark excitons and allows their hybridization with defect levels.^29^ These effects create a new electron–hole pair configuration known as an intervalley defect bright exciton, resulting in an efficient radiative decay.^27^ Furthermore, the emission from these defect-bound excitons occurs in pairs (doublets) with orthogonal linear polarizations.^10,25,30,31^ However, further studies are necessary to confirm the intervalley excitons model^27^ as the source of SPEs in WSe_2_.

Magneto-photoluminescence (magneto-PL) has turned out to be a useful technique to investigate the exciton and valley properties of 2D materials^32^ and could be used to probe the nature of SPEs.^9,11,12,18,25,33^ In fact, under a perpendicular magnetic field, a Zeeman splitting of the electronic and excitonic states is expected, where the associated *g*-factors depend on the nature of the emission peaks.^34,35^ For example, their values are around −4 for bright excitons, −8 for spin-forbidden dark excitons, and −13 for momentum-forbidden dark excitons.^34−36^ Under parallel magnetic field, the situation is quite different, as the magnetic field is expected to induce a mixing of the spin-up and spin-down states of electrons and holes.^35^ Furthermore, the parallel magnetic field also induces a splitting between the bright and dark excitons, which has been predicted and observed for MoSe_2_.^37,38^ On the other hand, no significant change on the PL peak energy has been detected for WSe_2_ monolayers under a parallel magnetic field^39,40^ up to ≈30 T, since the splitting of dark and bright excitons under a parallel magnetic field is expected to be inversely proportional to the zero-field separation of bright and dark excitons, which is much higher for WSe_2_ as compared with MoSe_2_.^37,38^ However, as we will show in this paper, the situation is different in the presence of local strain and defect levels.

Although there are several previous studies of magneto-PL under a perpendicular magnetic field for SPEs in WSe_2_,^9−12,18,24,25,33,41^ experiments under parallel magnetic field configuration are still elusive. Here, we investigate the nature of SPEs in WSe_2_ ML by using micro-photoluminescence (μ-PL) and magneto-photoluminescence (μ-magneto-PL) techniques under in- and out-of-plane magnetic fields. We studied samples of WSe_2_ ML on undoped (reference sample) and on 16%, in mol, of Tb_4_O_7_-doped borogermanate glass (BGB) substrates with different nanoroughness profiles. Second-order photon autocorrelation function measurements were also performed, and the antibunching behavior of SPEs was confirmed. Our findings indicate that altering the substrate doping impacts the strain profile, leading to an increased density of exciton doublets and enhanced SPE PL intensities. Additionally, we observed high values of the *g*-factor for SPEs in the presence of out-of-plane magnetic fields. Moreover, an anomalous redshift of SPE PL energies without any significant change in PL intensity was observed under an increasing in-plane magnetic field, which suggests that these SPEs are intervalley defect excitons. Furthermore, these results allow us to retrieve information about the exciton exchange interaction energies involved in the SPE process.

Our samples consist of WSe_2_ monolayers (MLs) on polished BGB glass. More details about the sample fabrication methods can be found in the Supporting Information (SI). The samples are schematically shown in Figure 1(a). The BGB substrate induces a random strain distribution in the WSe_2_ monolayer deposited on it, thus generating several localized excitons as illustrated in Figure 1.

**Figure 1:**
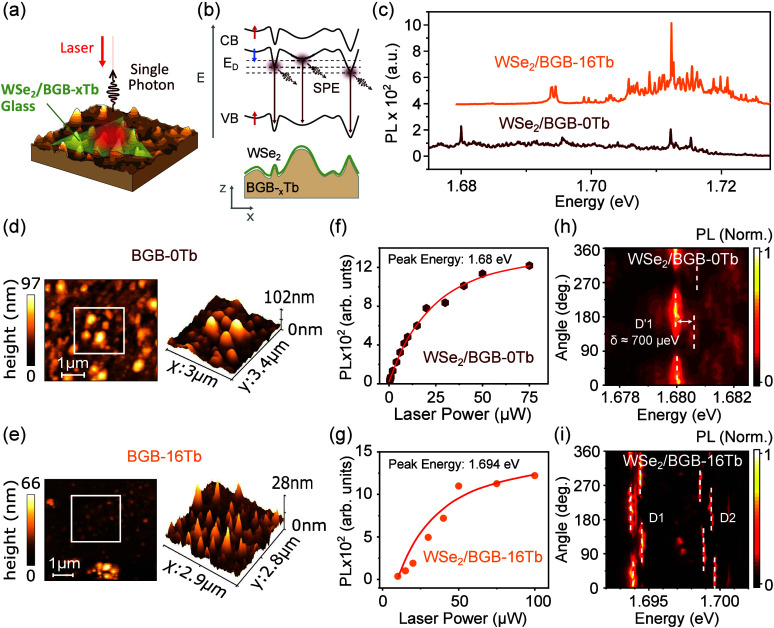
(a) Schematic representation of the sample with a WSe_2_ monolayer on polished glass, under laser excitation and showing the emission of single photons. (b) Schematic diagram of the conduction (CB) and valence (VB) band edges of *K*/*K*′ valleys. Under local strain, the band edges are deformed and confine excitons that can hybridize with defect levels (*E*_*D*_). The interaction of the confined dark excitons with the defect states generates a single-photon emission. (c) Typical PL spectra of WSe_2_ monolayers on BGB-16Tb and BGB-0Tb at temperature *T* = 3.6 K. Several sharp PL peaks are observed for both samples. (d, e) AFM topography image of the glass substrates (0% and 16% Tb^3+^). The Tb^3+^ doping affects the topology of the glass after polishing. (f, g) Typical laser power dependencies of PL intensity for the peak at 1.68 eV of the BGB-0Tb glass sample and 1.694 eV of the sample on BGB-16Tb glass, showing a saturation behavior. The solid red lines are a guide for the eyes. All sharp emission peaks show similar saturation behavior. (h, i) Color-coded map of the linearly polarized emission intensity as a function of the angle of in-plane polarization. The same fluctuation was observed for the emissions of each doublet, suggesting that they originate from the same QD. These doublets are separated by δ ≈ 700 μeV.

Figure 1(b) shows a schematic diagram of the conduction (CB) and valence (VB) band edges in the *K*/*K*′ valleys along the WSe_2_ ML. Under local strain, the band edges shift and the excitons can be strain-localized. Depending on the defect level position, a hybridization with these defect states (*E*_*D*_) may occur. The degree of band deformation depends on the strain profile and is, therefore, dependent on the laser’s position. The complexity of the strain field along the TMD plane results in different levels of hybridization, with deeper or shallower defects following the steepness of the strain field profile. Although several point defect types have been identified in 2D TMDs as potential sources of SPEs,^22,42−46^ any defect that breaks the valley symmetry and results in a localized state near the conduction band edge could play a similar role by allowing hybridization under strain.^27^

We have performed a detailed study of low-temperature μ-magneto-PL measurements under out-of-plane (Faraday configuration) and in-plane (Voigt configuration) magnetic fields to investigate the nature of localized excitons in the WSe_2_ ML. Figure 1(c) displays the typical PL spectra of the WSe_2_ ML on BGB glass substrates with and without Tb^3+^ doping at low temperatures (*T* = 3.6 K). The laser has spot size of 1 μm. The PL spectra on different sample positions show several sharp emission peaks (Figure S1). The sharp peaks have line widths between 165 μeV and 1 meV for the BGB-0Tb (0% of Tb^3+^) sample and 170 to 845 μeV for the BGB-16Tb sample (16% of Tb^3+^). Similar PL peaks were also observed in other systems such as nitrogen-diluted III–V compounds^47^ and oxygen-diluted II–VI compounds^48^ and were attributed to localized excitons. Remarkably, we have observed that the PL intensity of the Tb-doped sample (BGB-16Tb) is, on average, about two times higher than that for the undoped sample and features an increased number of sharp peaks. These two observations indicate stronger hybridization between the CB and defect levels (*E*_*D*_) (see Figure S1).

To understand the effects of substrate on SPE formation, we next investigated the substrate morphology using atomic force microscopy (AFM). Figures 1(d) and 1(e) show the AFM results for both the doped and undoped samples, respectively. We observe that the Tb^3+^ doping changes the morphology of the nanoroughness of polished glass substrates. The BGB-0Tb sample shows broader, rounded pillars with a height of ≈100 nm, while the BGB-16Tb sample has shorter, sharp pillars of ≈30 nm height. We also observed a higher density of sharp pillars for the doped glass substrate, which also exhibits a high density of sharp PL peaks.

Figures 1(f) and 1(g) illustrate the laser power dependence of the intensity of typical PL peaks, showing a saturation behavior characteristic of localized excitons. Measurements of the second-order correlation function (see Figure S5 in SI) show the antibunching behavior (*g*^(2)^(0) ≈ 0.3), demonstrating single-photon emission.^49^ Moreover, Figures 1(h) and 1(i) depict color maps of PL measurements as a function of linear polarization angle, revealing emissions occurring in pairs (doublets) with distinct linear polarization dependencies and the same spectral wandering (Figures S3 and S4, in SI). The observed doublets show an energy separation of ≈700 μeV, consistent with prior values reported in the literature.^9,10,18,20,21,25,30,31^ The presence of multiple sharp peaks in the experiments, spanning an energy range of 1.62 to 1.73 eV, is directly related to the substrate’s morphology. These PL peaks are associated with localized intervalley defect excitons induced by a complex strain field on the glass substrate (Figures 1(d) and 1(e)).

Figure 2 shows a schematic drawing of the sample and PL under Faraday configuration, i.e., perpendicular magnetic field . The color code map of σ^+^ circular polarization-resolved-PL intensity as a function of magnetic field for linearly polarized laser excitation at 3.6 K is shown in Figures 2(a) and 2(b) in the range of −6 to 6 T. The negative values of magnetic fields are equivalent to the σ^–^ component due to time-reversal symmetry. Doublet structures can be well identified, such as the doublets labeled D′1–D′3 for WSe_2_/BGB-0Tb and D1–D6 for BGB-16Tb. Notably, a stronger valley polarization degree for the σ^+^ component is observed for both samples.

**Figure 2:**
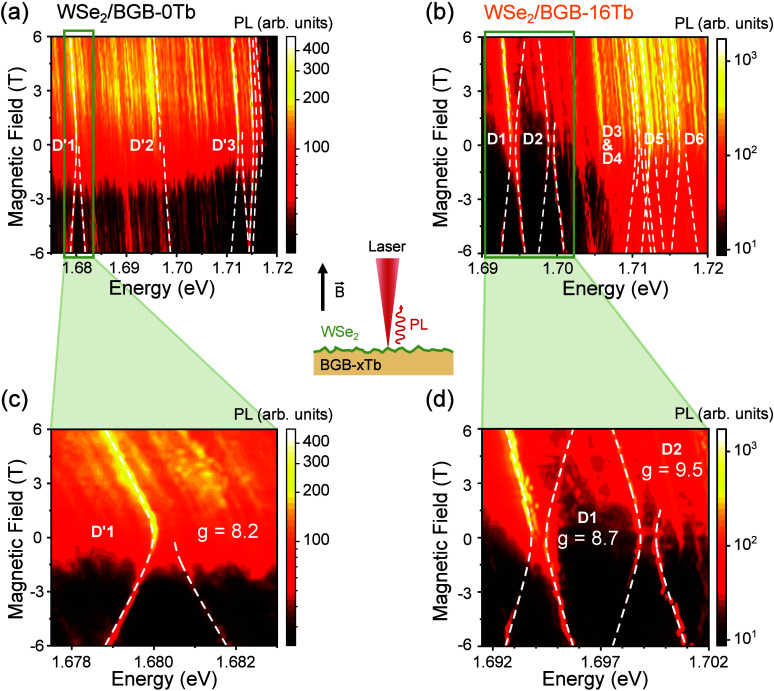
μ-PL measurement under a magnetic field applied perpendicular to the plane of the WSe_2_ monolayer, which is deposited over a BGB substrate doped with *x* percent of Tb (schematic drawing in center). (a, b) Color maps of the circularly polarized PL spectra as a function of the magnetic field for the WSe_2_ samples on glass with 0% and 16% Tb^3+^, respectively. The sample was excited with a linearly polarized laser, and the σ^+^ component was collected. We note that SPE emissions for both samples are strongly polarized for positive magnetic fields. The PL intensity of the WSe_2_/BGB-16Tb sample, at the same experimental conditions, was about 3× stronger, and they showed more distinguishable doublets than the WSe_2_/BGB-0Tb sample. (c, d) Portion of the results around the D′1 (WSe_2_/BGB-0Tb) and D1 and D2 (WSe_2_/BGB-16Tb) doublet regions observed in (a) and (b), with their respective effective *g*-factors. The energy shift for each doublet branch was fitted using eq 1.

The details of the magnetic field dependence of the PL spectra for the D′1 (BGB-0Tb), D1, and D2 (BGB-16Tb) doublets are observed in Figures 2(a) and 2(b) and are magnified in Figures 2(c) and 2(d). A clear anticrossing behavior is observed in the energy spectra of PL peaks close to zero field. In order to extract the *g*-factors of the excitons involved in this doublet, the Zeeman shifts λ_⊥,±_ have been fitted using the following equation:^9,10,18,27,33,50^
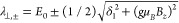
1which is inferred from the theoretical model for these exciton doublets under an out-of-plane magnetic field, explained in detail in the SI. Here, *g* is the effective *g*-factor, μ_*B*_ is the Bohr magneton, and δ_1_ is the zero-field-splitting fine structure due to exchange interactions between excitons involving defect states with opposite spins. The white dashed lines in Figures 2(c) and 2(d) highlight the magnetic field dependence of the peaks. The *g*-factors of bright excitons and trions are expected to have typical values of *g* ≈ −4, according to previous experiments^51−55^ and theoretical predictions.^56^ The *g*-factor for the dark exciton, however, has a theoretical expectation of −8,^57,58^ which is also consistent with previous experimental reports in the literature.^50,54,55,58,59^ Interestingly, the doublets presented here exhibit *g*-factors of *g* = 8.2 for D′1, *g* = 8.7 for D1, and *g* = 9.5 for D2 under an out-of-plane magnetic field, similar to the values of the dark exciton, although distinctly different due to local strain.^50^ Particularly, the magnetic field dependence of these sharp peaks indicates that its spin-valley configuration is almost identical to that of the dark exciton.^57^ It is worth noting that *g*-factors ranging from 2 to 13 for sharp peaks in WSe_2_ have also been reported in the literature and associated with different natures.^25,33,41^ Our results, however, support the evidence that this value of *g*-factors is a result of hybridization between defects and the dark states of WSe_2_. Around 36 peaks were analyzed in both samples, and most of them have similar behavior, with slightly different values for *g* and δ_1_, reinforcing the impact of a complex strain field. The summary of these values is presented in the SI (Figures S9 and S10).

The behavior of exciton doublets under the out-of-plane magnetic field observed here only informs us about one of the exchange energies involved in the exciton hybridization, characterized by the parameter δ_1_. However, considering the hybridization of exciton states involving spin-up and -down electrons confined by defects and *K*/*K*′ hole states, one ends up with four exciton eigenstates in the system.^27^ Only two of these exciton states are brightened by this hybridization, which are indeed observed as doublets in the experiment. The parameter δ_1_ represents the zero-field energy split between them, but, with results within the Faraday configuration, no additional information can be retrieved about the splitting with the other two states in the aforementioned set of four exciton eigenstates. As we will explain in what follows, this missing information is retrieved by investigating the dependence of the exciton doublet under an *in-plane* magnetic field, i.e., performing measurements within the Voigt configuration.

Let us now discuss the magneto-PL results under a magnetic field applied parallel to the materials plane (Voigt configuration). Figure 3 shows a schematic drawing and PL results for both samples under such a field. Under a parallel magnetic field, it is expected that the magnetic field acts in mixing the spin components of excitons in each valley, thus resulting in a brightening of the dark excitons^38−40^ and an energy shift of dark and bright states.^38^ Since the spin–orbit coupling is the effect behind the natural spin polarization at K/K′ valleys in TMDs, a small spin–orbit coupling is required to observe significant energy shifts at reasonable experimental values of an in-plane magnetic field. Indeed, this energy shift has been evidenced for MoSe_2_ML in several previous works.^37−39^ However, particularly for WSe_2_ monolayers, it has been reported that, due to their stronger spin–orbit coupling, the excitonic energy shifts for this material are negligible as compared to those of MoSe_2_.^37,40^ Furthermore, as we previously mentioned, the presence of local strain and defects results in the brightening of dark excitons and in doublet emission at zero magnetic field. Nevertheless, it is still unclear how in-plane magnetic fields would affect SPE properties in the WSe_2_ monolayer. This sparks our interest to investigate the magneto-PL of these SPE doublets under an in-plane magnetic field, in order to probe the nature of these peaks.

**Figure 3:**
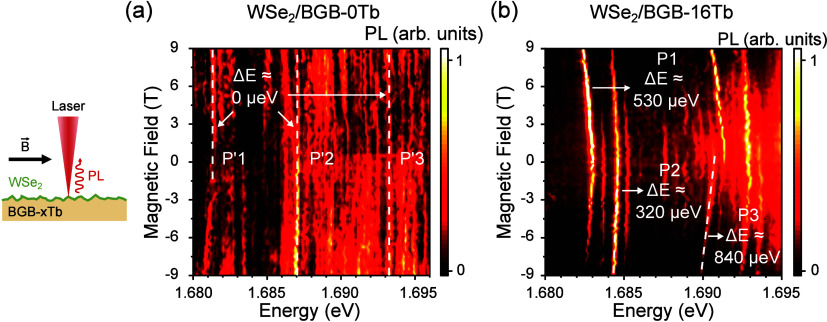
On the left, a schematic drawing of μ-PL measurement under a magnetic field applied parallel to the plane of the monolayer. (a, b) Color-coded map of the PL intensity as a function of the magnetic field for the 0% and 16% Tb^3+^ samples, respectively. Each color-coded plot was normalized by the maximum intensity. The magnitude of the peak position displacement with increasing magnetic field depends on the sample and laser position.

Figures 3(a) and 3(b) illustrate the color-coded map of PL intensity as a function of magnetic field under an in-plane magnetic field for both samples, BGB-0Tb and BGB-16Tb, respectively. We observed that the magneto-PL properties for different samples are clearly distinct. In the case of the undoped sample (BGB-0Tb) with smoother nanoroughness, most PL peaks display very small energy shifts. Conversely, for BGB-16Tb with a substrate featuring sharper nanoroughness profiles, most peaks show clear redshifts (of about 530–840 μeV) with increasing in-plane magnetic field. Remarkably, no significant increase in the intensity of these emissions was observed as the magnetic field was increased, in contrast to the previous expectations of spin mixing and exciton brightening under in-plane fields.

In the absence of local strain, theory predicts a negligible energy shift and enhancement of PL intensity for the dark excitons of monolayer WSe_2_ under increasing in-plane magnetic field.^38,40^ However, if we consider the mixing of localized dark exciton and intervalley defect excitonic states, then one can describe the available states at the band edges using a generic four-level Hamiltonian. An effective model, presented in the theoretical section of the SI, can then be used to predict the effect of in-plane magnetic fields on these energy levels. This model involves only the combinations between electrons in the lower energy conduction band and holes in the higher energy valence band at *K*/*K*′ valleys of WSe_2_, namely, the electron–hole pairs that are naturally dark in the absence of defects and strain. Defect localization relaxes selection rules and allows these electron states to form excitons with holes from both the *K* and *K*′ valleys. This results in four types of electron–hole pairs, whose degeneracy is broken by exchange interactions. Even after this exchange-induced hybridization of exciton states, a pair of higher energy eigenstates remains dark, separated by an energy δ_2_ from the lower energy bright exciton doublet, which is the doublet observed in our magneto-PL experiments.^27^ Results in Figure 3 show that both states in the doublet undergo a redshift as the magnetic field increases. Opposite to the conventional dark exciton emissions, their PL intensities do not significantly improve with the magnetic field, as those states have already been brightened by induced hybridization with defect states. Our calculations show that the redshift, as a function of the in-plane magnetic field *B*_∥_, follows
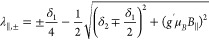
2The parameters are analogous to those of the effective Zeeman shift with perpendicular fields, but since orbital contributions to the angular momentum should not play a role for electrons and holes under in-plane magnetic field, the *g*′-factor here is assumed to have a major contribution from the spin component, thus resulting in *g*′ ≈ 2. Also, δ_2_ is a parameter characterizing the hybridization between the strain-confined and intervalley defect excitonic states, which results in splitting between the bright exciton doublet and the higher energy dark exciton doublet. Their values are about 1 meV for samples with stronger local strain in WSe_2_/BGB-16Tb (see Figures S11). For the BGB-0Tb sample, the negligible PL peak redshifts indicate that the smoother surface only weakly hybridizes with the defect states. However, for the BGB-16Tb sample, the experimentally observed PL peak redshifts of the bright doublet under Voigt configuration allow us to use eq 2 to infer a δ_2_ ≈ 1 meV for the exchange-induced splitting, which agrees well with density functional theory (DFT) predictions of this energy,^27^ but has not been experimentally probed so far, to the best of our knowledge.

In conclusion, we have investigated the nature of SPEs in WSe_2_ monolayer samples with different nanoroughness profiles by magneto-PL measurements under in-plane and out-of-plane magnetic fields. Several sharp PL peaks were observed and are identified as excitonic doublet SPEs. These PL peaks are stable over time and show well-defined linear light polarization. We found a significant enhancement of the density of doublets and PL intensity with an increasing local strain profile. These PL peaks also reveal high values of effective *g*-factors, between 6 and 11. Notably, we observed an unexpected redshift in the energy of PL peaks by increasing the magnitude of an in-plane magnetic field, which strongly depends on the local strain profile. The observed redshift of PL energy peaks and lack of change of PL intensity with increasing magnetic field for the sharp PL peaks are explained by the brightening of dark excitons due to the hybridization of defect levels and strain-localized dark excitons. Furthermore, such a redshift allowed us to experimentally probe the magnitude of exchange interaction energies between excitonic states in the system in the presence of strain and defects. We present a model to explain these results. The values of these exchange interactions are extracted and are around 1 meV depending on the local strain profile. Our work provides a comprehensive discussion of the nature of single-photon emitters in WSe_2_ ML and more efficient control of SPEs, paving the way for future practical integration of 2D materials into quantum information systems.

## Supporting Information Available

Details of sample preparation; complementary PL data for the characterization the SPEs in WSe_2_/BGB-0Tb and WSe_2_/BGB-16Tb samples; measurement of the second-order photon autocorrelation function; PLE data for both samples; complementary magneto-PL data and summary of the extracted effective *g*-factors; theoretical model (PDF)
