# Supplementary material for: Probing the Nature of Single-Photon Emitters in a WSe2 Monolayer by Magneto-Photoluminescence Spectroscopy
Source: Nano Lett. 2024 Oct 10;24(42):13300–6. doi: 10.1021/acs.nanolett.4c03686 (PMC11503778; doi:10.1021/acs.nanolett.4c03686)
Supplement: Supplementary file 1 — nl4c03686_si_001.pdf [file nl4c03686_si_001.pdf]

Probing the nature of single-photon emitters in WSe_2 monolayer by magneto-photoluminescence spectroscopy

Caique Serati de Brito,[†] Bárbara L. T. Rosa,[‡] Andrey Chaves,[¶,§] Camila Cavalini,[†] César R. Rabahi,[†] Douglas F. Franco,[∥] Marcelo Nalin,[∥] Ingrid D. Barcelos,[⊥] Stephan Reitzenstein,[‡] and Yara Galvão Gobato[*,†]

†*Department of Physics, Federal University of São Carlos, São Carlos, SP, 13565-905, Brazil*

‡*Institute of Solid State Physics, Technische Universität Berlin, 10623 Berlin, Germany*

¶*Universidade Federal do Ceará, Departamento de Física, 60455-760 Fortaleza, Ceará, Brazil*

§*Department of Physics & NANOlab Center of Excellence, University of Antwerp, Groenenborgerlaan 171, B-2020, Antwerp, Belgium*

∥*Institute of Chemistry, São Paulo State University—UNESP, CEP 14800-060 Araraquara, SP, Brazil*

⊥*Brazilian Synchrotron Light Laboratory (LNLS), Brazilian Center for Research in Energy and Materials, Campinas (CNPEM), Campinas, SP, Brazil*

E-mail: yara@df.ufscar.br

Samples Preparation

The borogermanate (BGB) glass substrates were prepared by the melt-quenching method using the following precursors: germanium oxide (GeO_2, Sigma-Aldrich), boric acid (H_3BO_3, Sigma-Aldrich), sodium carbonate (Na_2CO_3, Sigma-Aldrich), barium carbonate ($BaCO_3$, Sigma-Aldrich), aluminum oxide (Al_2O_3, Sigma-Aldrich), and terbium oxide (Tb_4O_7, Sigma-Aldrich).

They consist of molar compositions of $41GeO_2$–$25B_2O_3$–$4Al_2O_3$–$10Na_2O$–$20BaO$ and $83.75(41GeO_2$–$25B_2O_3$–$4Al_2O_3$–$10Na_2O$–$20BaO)$–$16Tb_4O_7$–$0.25Ce_2O_3$. The samples were labeled as BGB-0Tb and BGB-16Tb, respectively. Initially, the chemicals were stoichiometrically weighed to yield 7 g. Subsequently, they were ground and homogenized into a fine powder using a Flack Tek speedmixer equipment for 2 min. The glass compositions BGB-0Tb and BGB-16Tb were loaded into a platinum crucible and melted at 1250 °C and 1500 °C, respectively for 2 h, under atmospheric conditions. After melting, the glass was cooled in a preheated steel mold at 50 °C below the glass transition (Tg) temperature and then annealed for 5 hours until it reached room temperature. The Tg values were 549 °C and 733 °C for BGB-0Tb and BGB-16Tb, respectively. As a result, bulk glasses with a thickness of 3 mm were obtained. The glasses were then polished in several stages using silicon carbide (SiC) polishing papers with various granulometries, including 600, 800, 1000, 1200, 2400, and 4000 mesh. As a final step, the glasses were polished with an alumina suspension with granulometry of 0.50 μm.

The 2D samples were prepared by conventional all-dry transfer techniques and consist of a ML of WSe_2 (bulk crystals from HQ Graphene) on a borogermanate polished glass. The WSe_2 MLs were first exfoliated by Scotch tape and later exfoliated again on a polydimethylsiloxane (PDMS) stamp and placed on a glass slide for inspection with an optical microscope and transferred to the borogermanate glass substrate.

The samples were mounted on attocube piezoelectric x–y–z translation stages to control the sample position. The x-y-z stage was then placed inside a helium closed-cycle cryostat

with a superconducting magnet coil (attocube—Attodry1000) capable of generating magnetic fields up to 9 T, perpendicular (Faraday configuration) or parallel (Voigt configuration). The μ-photoluminescence (PL) measurements were performed using a continuous-wave (CW) laser with a photon energy of 1.879 eV (660 nm). The laser was focused on the sample by an aspheric lens (NA = 0.68), with a spot size of about 1 μm. All μ-PL measurements were performed with a laser power of 20 μW (otherwise when specified) with the sample inside an insert and cooled by helium exchange gas, ensuring minimal sample heating during the experiments. The laser excitation is linearly polarized and the magneto-PL detection is circularly polarized. The polarization of the PL signal was selected before being focused into a 5 μm single-mode optical fiber. The signal was then dispersed by a 75 cm spectrometer with a 1200 l/mm and 150 l/mm grating and detected by a silicon CCD detector (Andor, Shamrock/iDus).

PL Results

We have investigated in detail the optical properties of the WSe_2/BGB glass samples. Below, we present the complementary optical results, highlighting various aspects of the PL emission.

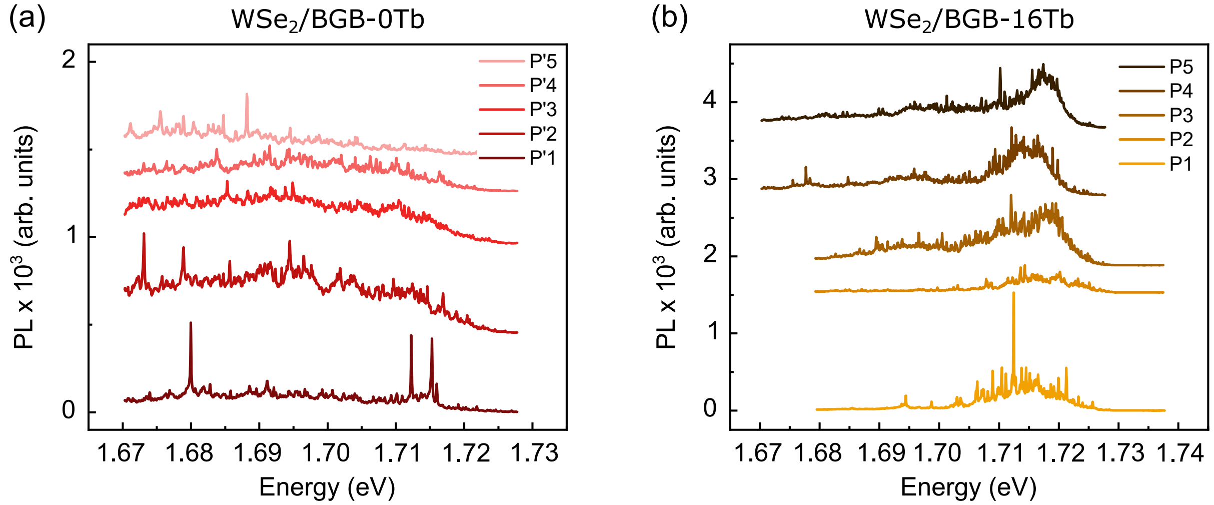

Fig. S1: PL spectra for different laser positions on WSe_2/BGB-0Tb and WSe_2/BGB-16Tb samples at 3.6K.

Figure S1 displays the PL spectra for the WSe_2/BGB-0Tb and WSe_2/BGB-16Tb samples at 3.6 K using a grating of 1200 l/mm. The laser power was 20 μW. Several sharp PL peaks were observed and attributed to SPEs. We observe that the PL peak positions are different for different laser position on the sample. For the WSe_2/BGB-0Tb sample, the PL peaks appear over a broader energy range (1.67-1.72 eV). However, these sharp peaks are weakly pronounced. In contrast, the WSe_2/BGB-16Tb sample exhibits a large number of sharp peaks in the 1.695-1.725 eV range, with higher intensity (their intensity is approximately three times the PL intensity of WSe_2/BGB-0Tb reference sample) and a noticeable broadband background emission in this range. We averaged the emission intensity of the ten most intense peaks from each measured position to provide a reliable comparison of photoluminescence intensities between the BGB-16Tb and BGB-0Tb samples. This approach allows for a consistent comparison across all positions. The analysis reveals an intensity ratio of approximately two between the BGB-16Tb and BGB-0Tb samples, as shown in Figs. S1(a) and (b). This result, combined with the increased density of sharp peaks in the PL spectra, supports the conclusion of stronger hybridization between the conduction band and defect levels. These observations indicate that a greater number of localized intervalley defect excitonic states are becoming optically active, contributing to the enhanced PL intensity in the Tb-doped sample.

The PL spectra for both samples, using a 150 l/mm grating, are presented in Figure S2. The exciton energy peak is observed at approximately 1.75 eV in the WSe_2/BGB-0Tb sample while for the WSe_2/BGB-16Tb sample is highly quenched and centered at higher energy at around 1.765 eV. The sharp emission peaks in WSe_2/BGB-16Tb sample are observed at lower energies as compared to the reference sample. The PL peak energies lie between 35 meV and 127 meV below the exciton peak (X), and for the WSe_2/BGB-0Tb sample and between 46 meV and 73 meV for the WSe_2/BGB-16Tb sample. These results show that the sharp PL peaks for the WSe_2/BGB-0Tb are mainly observed at a lower energy range as compared to WSe_2/BGB-16Tb due to different local strain profiles for different samples.

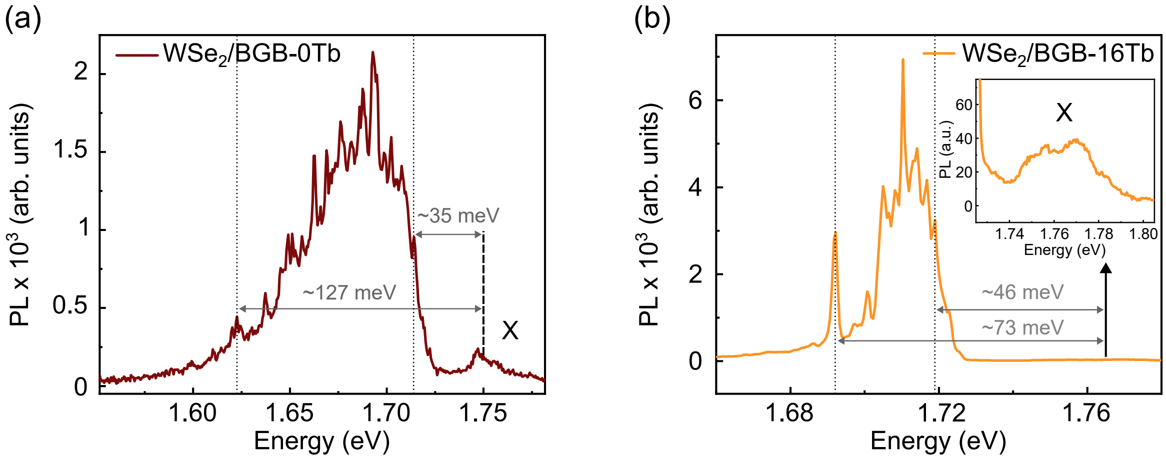

Fig. S2: PL spectra for the WSe_2/BGB-0Tb and WSe_2/BGB-16Tb samples at 3.6K, using a grating of 150 l/mm.

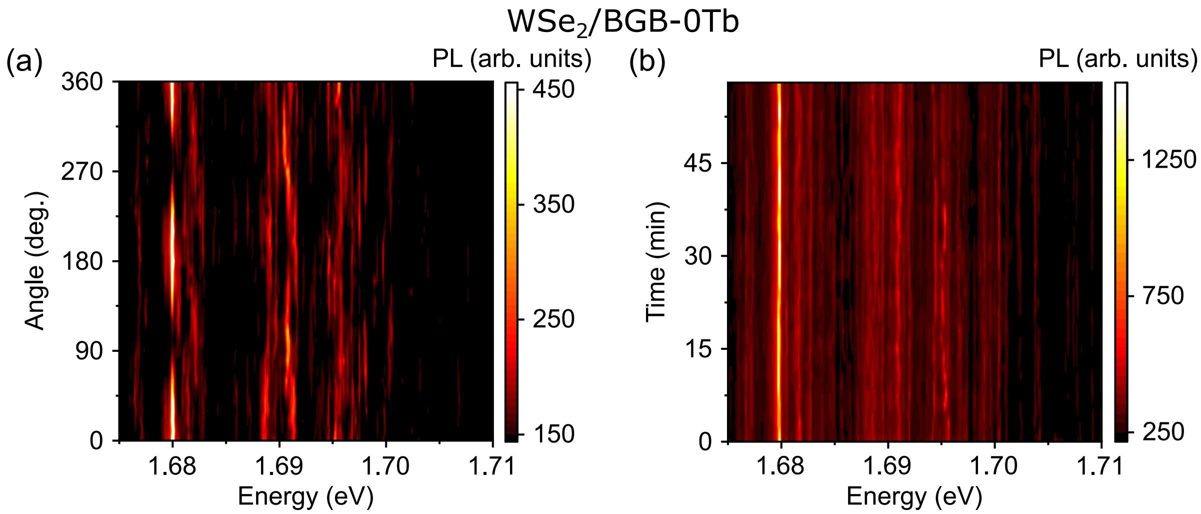

Fig. S3: (a) Color-coded map of the linearly-polarized emission intensity as a function of the angle of in-plane polarization. (b) Color-coded map of the time dependence of the μ-PL emission feature showing jittering effects.

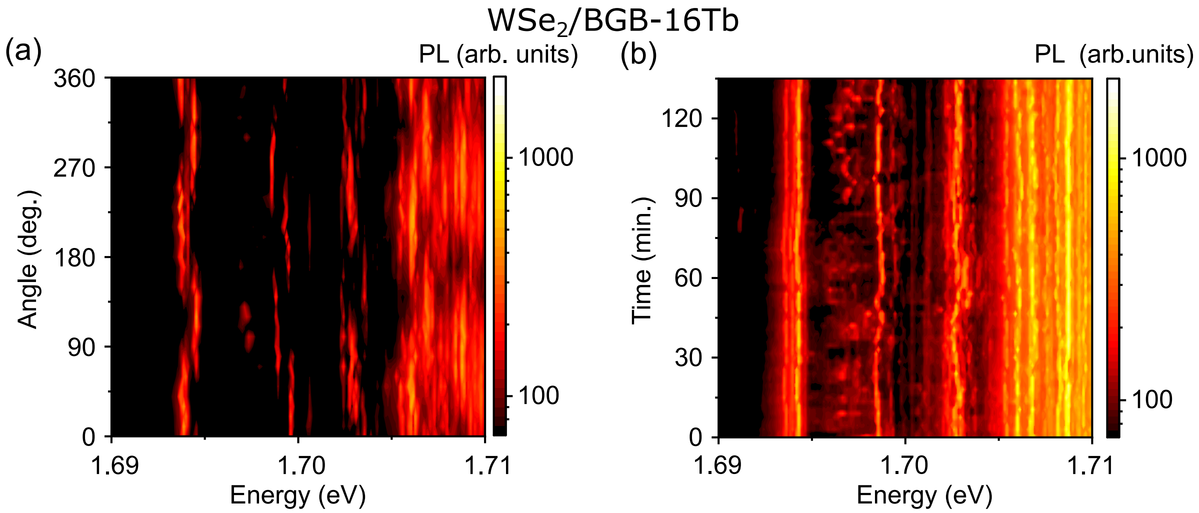

Fig. S4: (a) Color-coded map of the linearly-polarized emission intensity as a function of the angle of in-plane polarization. (b) Color-coded map of the time dependence of the μ-PL emission feature showing jittering effects. The doublets can be identified either by their orthogonal dependence in linear polarization or spectral wandering over time.

We have also performed measurements of linearly polarized resolved PL as a function of the angle of in-plane polarization. A half-wave plate and a linear polarizer were used for the linear polarization detection. Figures S3 and S4 show the linearly polarized resolved PL for both samples.

The time stability of the PL was also investigated. The PL signals were obtained for 90 s intervals over a period of more than 1 h. We observed that the PL signal of the sharp peaks have good stability on time. Usually, SPEs related to the presence of a non-uniform strain and defects manifest as doublets with orthogonal linear polarization and have similar spectral wandering over time. Figures S3 and S4 shows the doublets D'1, D1 and D2 presented in the main text which are identified by the characteristics mentioned above.

In order to evidence the SPE´s nature of the observed sharp peaks, we measured the second-order photon auto-correlation function in the WSe_2/BGB-0Tb sample. The experimental configuration consists of a CW 660 nm laser for excitation and Semrock tunable short- and long-pass optical filters to spectrally select the emission peak of interest, as shown in Figure S5(a). The emission was coupled into a fiber-based Hanbury–Brown and Twiss

setup with two superconducting nanowire single-photon detectors (SNSPDs). Figure S5(b) displays the measured auto-correlation function for an emission at 1.626 eV (763 nm) at $(\tau) = 0$ of $g^{(2)}(0) = 0.32 \pm 0.02$, which lies below 0.5 and therefore confirms the photon anti-bunching signature of single-photon emitters.

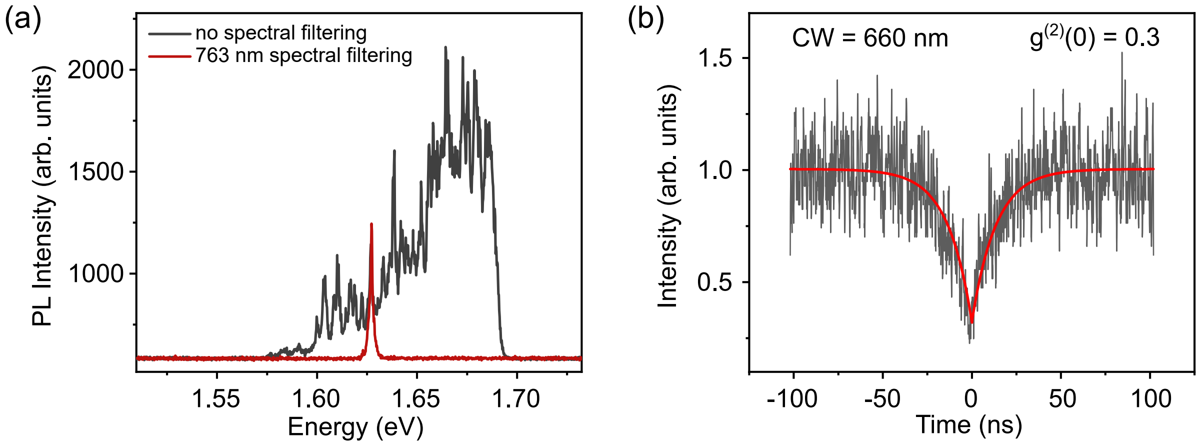

Fig. S5: (a) PL Spectra of the WSe_2/BGB-0Tb. In red the selected peak for the analysis. (b) Second-order correlation function $g^{(2)}$ as a function of time delay measured for a PL peak at 1.626 eV at the WSe_2/BGB-0Tb sample. The red curve shows the fit after deconvolutions of the second order correlation function. The extracted zero delay $g^{(2)}(0)$ is 0.3. The $g^{(2)}(0)$ function shows clear anti-bunching behavior which is the fingerprint of SPEs.

Photoluminescence excitation (PLE) spectroscopy was also measured for both samples. PLE provides detailed information about the absorption properties and electronic structure of SPEs in WSe_2 as it can reveal the resonant absorption peaks corresponding to different excitons and their coupling to defect states or localized excitons responsible for single-photon emitters generation. We extracted the intensity of a broad PL emission range that shows several single-photon emitter candidates while exciting the sample from 1.675 to 1.758 eV using a picosecond tunable optical parametric oscillator (OPO) laser. The highest energy excitation coincides with the neutral exciton emission and, therefore, it cannot be observed in Figures S6 and S7. The PLE response of transition metal dichalcogenides(TMD)-based SPEs has been reported in the literature previously and shows distinguishable results. These results vary from: (i) a clear resonance energy separation resembling a typical semiconductor quantum dot, where discrete energy levels of the confinement potential are observed;[1] and

(ii) absorption resonances of SPEs located in the excitonic complex region, as seen in the monolayer WSe_2 with no apparent localized excitons.[2]

For the sample WSe_2/BGB-0Tb, we predominantly observe that the PLE resonances of three emitters with different emission energies are associated with excitonic complexes of WSe_2, as in the aforementioned situation (ii).[2] However, for the sample, WSe_2/BGB-16Tb, the PLE resonance of the similar three analyzed peaks appears even more intense at the bright neutral exciton peak (X).

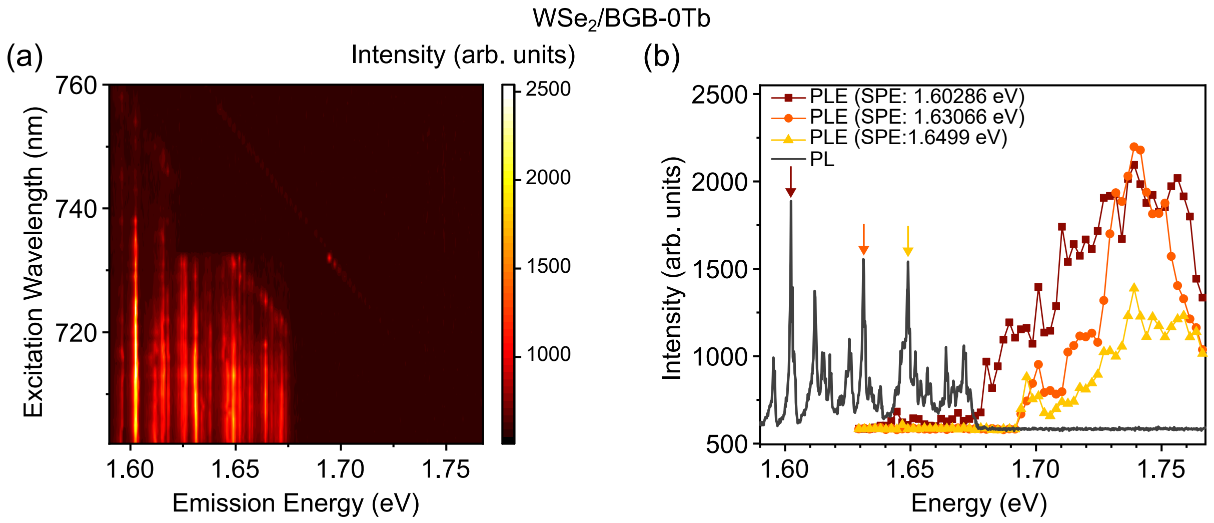

Fig. S6: (a) Color-coded map of the PLE intensity for the WSe_2/BGB-0Tb sample at 4K. (b) Typical PL spectra and intensity as a function of the excitation energy for the selected peaks.

Figure S8 shows the color-coded map of circularly resolved PL intensity under out-of-plane magnetic field up to 6 T for both samples for the energy range of 1.71 eV to 1.78 eV. The samples were excited with linearly polarized light, and a quarter-wave plate was set to select the σ^+ component with positive magnetic fields. Due to time-reversal symmetry, the negative values of the field correspond to the σ^- component. We clearly observed that the WSe_2/BGB-0Tb and WSe_2/BGB-16Tb samples show different densities of doublets for the same spectral region.

For the WSe_2/BGB-0Tb sample (see Figure S8(a)), we found that the peaks exhibit a blue shift much less intense, making it more challenging to correctly identify doublets. All

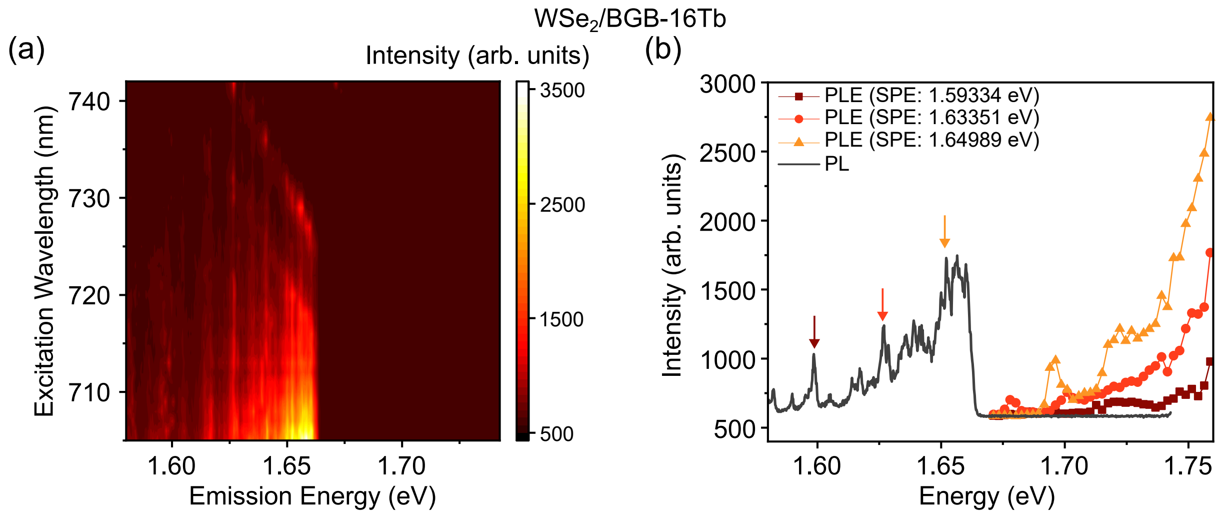

Fig. S7: (a) Color-coded map of the PLE intensity for the WSe_2/BGB-16Tb sample at 4K. (b) Typical PL spectra and intensity as a function of the excitation energy for the selected peaks.

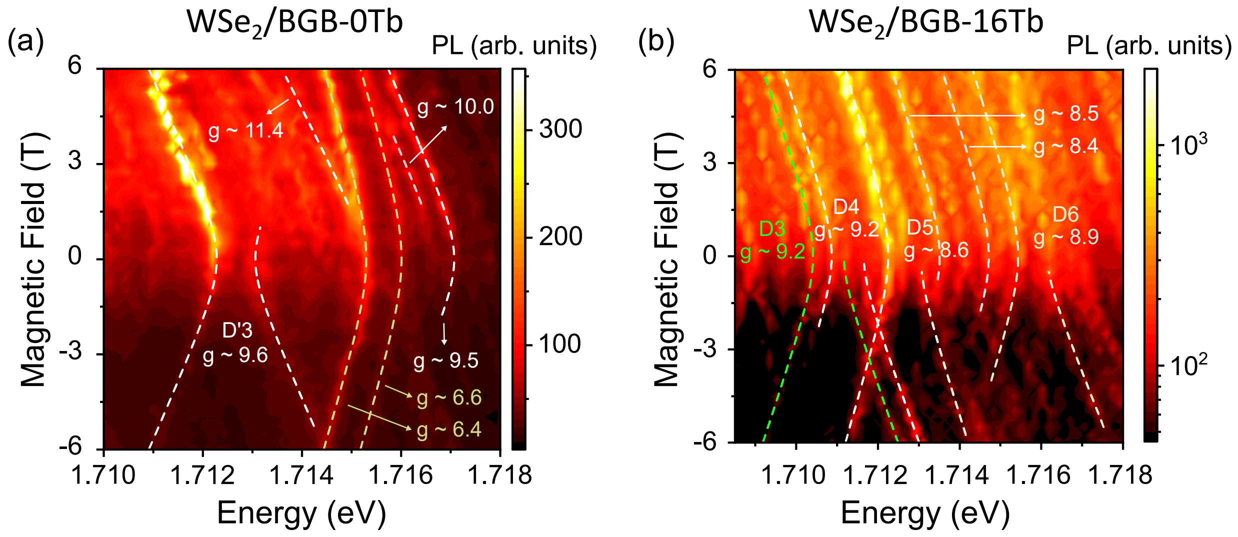

Fig. S8: Color-code map of the circularly resolved μ-PL intensity as a function of out-of-plane magnetic field, at 3.6 K, for the WSe_2/BGB-0Tb (a) and WSe_2/BGB-16Tb (b) samples. We observed that the samples show a distinct density of SPE.

peaks are fitted by Eq. (1) of the main text: $\lambda_{\perp,\pm} = E_0 \pm \frac{1}{2}\sqrt{\delta_1^2 + (g\mu_B B_z)^2}$. The theory leading to this equation is explained in details further on, in the following Section. Most of the sharp PL peaks displayed higher g-factors, ranging from 9.5 to 11.4, consistent with values reported in the literature.[3] Additionally, we also observed few PL peaks with lower g-factor values, such as $g = 6.4$ and $g = 6.6$, which could be associated with different types of single-photon sources. These peaks may be linked to optical transitions involving defect levels.[4]

In contrast, several doublets were identified for the sample WSe_2/BGB-16Tb. Figure S8(b) shows the typical doublets observed for this sample, labeled as D3 to D6. In this range, all PL peaks exhibited g-factors between 8.4 and 9.2. Other peaks showing similar behavior related to the out-of-plane magnetic field can also be observed in this region.

Figures S9 and S10 show the color-coded map of the PL intensity as a function of the perpendicular magnetic field up to 9 T for the WSe_2/BGB-0Tb and WSe_2/BGB-16Tb samples, respectively. The spectra from both samples feature many sharp peaks. To better understand their behavior, we took the spectra in 0.25 T intervals, sweeping the magnetic field from 6 T to -6 T. For each spectrum, we extracted various peak positions based on their maximum intensity. The analyzed peaks are labeled and presented in the tables of Figures S9 and S10. These points, covering the measured magnetic fields, were then fitted using Equation (1). In order to cover both cases of the equation, we used the index j $= \pm\frac{1}{2}$ (in the tables), to characterize the blue and redshift of the peaks. By comparing the E_0 values, i.e., the center point between doublets, of counter-shifting peaks, we were able to identify the pairs coming from the same emitter. The g-factors and zero-field splitting fine structure values for different PL peaks are shown in the tables on the right side of Figures S9 and S10. The error bars were estimated from the fittings and are affected by the jittering of the peaks, the proximity of neighboring peaks depending on the magnetic field, and reduced PL intensity under negative magnetic fields.

Under in-plane magnetic fields, we observed a clear redshift of PL peaks for the WSe_2/BGB-

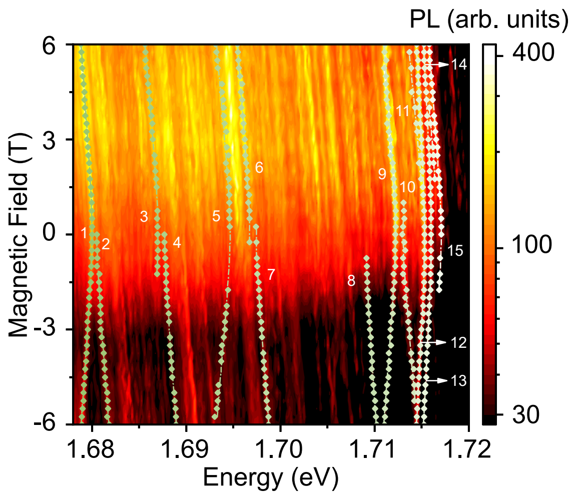

WSe$_2$/BGB-0Tb					
Label		$E_0 \pm \Delta E_0$ (eV)	j	$\delta_1 \pm \Delta\delta_1$ (μeV)	$g \pm \Delta g$
1	D'1	1.68027 ± 2E-05	0.5	620 ± 50	8.2 ± 0.2
2		1.68030 ± 1E-05	-0.5	600 ± 20	8.2 ± 0.1
3		1.68735 ± 2E-05	-0.5	710 ± 40	9.4 ± 0.1
4		1.68735 ± 1E-05	0.5	680 ± 30	9.1 ± 0.1
5		1.69500 ± 2E-05	-0.5	580 ± 80	10.1 ± 0.2
6	D'2	1.69707 ± 1E-05	-0.5	690 ± 50	9.2 ± 0.1
7		1.69709 ± 1E-05	0.5	690 ± 30	9.4 ± 0.1
8		1.70872 ± 1E-05	0.5	710 ± 40	8.0 ± 0.1
9	D'3	1.71265 ± 1E-05	-0.5	680 ± 60	9.4 ± 0.1
10		1.71267 ± 2E-05	0.5	650 ± 60	10.0 ± 0.2
11		1.71560 ± 3E-05	-0.5	720 ± 30	11.4 ± 0.4
12		1.71564 ± 1E-05	-0.5	700 ± 30	6.6 ± 0.1
13		1.71635 ± 2E-05	-0.5	690 ± 20	6.4 ± 0.1
14		1.71703 ± 7E-05	-0.5	700 ± 100	10.0 ± 0.4
15		1.71742 ± 1E-05	-0.5	710 ± 30	9.5 ± 0.1

Fig. S9: Color-code map of the circularly resolved μ-PL intensity as a function of positive and negative out-of-plane magnetic field, at 3.6 K, for the WSe$_2$/BGB-0Tb sample. The peaks were fitted using Eq.(1) in the main text. A summary of the fitting parameters for different sharp peaks is shown in the table on the right side. In addition to spectral wandering and linear polarization, the doublets can also be assigned by the similarity in the obtained parameters. The index j indicates if the peak is red or blue-shifting with the increased magnetic field

16Tb sample. In Figure S11 we present the extracted energy positions of the peaks P1 and P2 in Figure 3(b) of the main text, which ranged from 9 T to -9 T of magnetic fields. We can test our model using Equation (2) of the main manuscript to estimate the exchange interaction δ_2. We extracted the parameter δ_2 based on the expected effective g-factor of about $g' = 2$, allowing small variations due to strain effect, and using $\delta_1 = 700$ μeV, as averaged from the values obtained here and in other studies. For the WSe$_2$/BGB-16Tb, the peaks consistently redshift with the increase in the strength of the magnetic field. Following our presented model, the δ_2 values are about ≈1 meV, in the same order of theoretical predicted values.[5] For the WSe$_2$/BGB-0Tb, only very small shifts in energy are observed, being negligible considering the spectral wandering of the peaks. This indicates a weak hybridization (large δ_2) deviating from the idealization of our model.

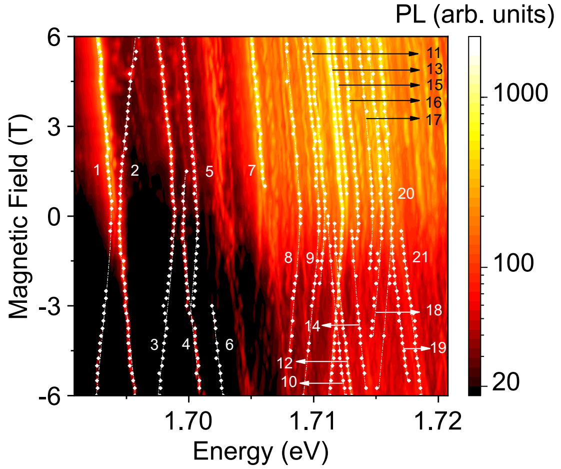

WSe$_2$/BGB-16Tb					
Label		$E_0 \pm \Delta E_0$ (eV)	j	$\delta_1 \pm \Delta\delta_1$ (μeV)	$g \pm \Delta g$
1	D1	1.69412 ± 1E-05	-0.5	670 ± 40	8.6 ± 0.1
2		1.69413 ± 1E-05	0.5	680 ± 50	8.8 ± 0.1
3	D2	1.69920 ± 1E-05	-0.5	730 ± 50	9.3 ± 0.1
4		1.69920 ± 2E-05	0.5	730 ± 50	9.8 ± 0.2
5		1.70096 ± 9E-05	0.5	700 ± 100	9.9 ± 0.6
6		1.70102 ± 3E-05	-0.5	700 ± 50	8.6 ± 0.3
7		1.70639 ± 2E-05	-0.5	690 ± 40	7.7 ± 0.2
8		1.70923 ± 3E-05	-0.5	680 ± 60	8.7 ± 0.4
9	D3	1.71078 ± 1E-05	-0.5	740 ± 30	9.1 ± 0.1
10		1.71080 ± 1E-05	0.5	730 ± 50	9.3 ± 0.1
11	D4	1.71120 ± 1E-05	-0.5	720 ± 40	9.1 ± 0.1
12		1.71131 ± 1E-05	0.5	710 ± 50	9.3 ± 0.1
13	D5	1.71268 ± 2E-05	-0.5	680 ± 40	8.7 ± 0.2
14		1.71271 ± 2E-05	0.5	690 ± 40	8.4 ± 0.2
15		1.71302 ± 2E-05	-0.5	690 ± 30	8.2 ± 0.2
16		1.71386 ± 2E-05	-0.5	680 ± 40	8.5 ± 0.2
17		1.71506 ± 1E-05	-0.5	700 ± 30	8.4 ± 0.2
18	D6	1.71578 ± 3E-05	-0.5	690 ± 60	8.2 ± 0.4
19		1.71580 ± 2E-05	0.5	710 ± 40	10.6 ± 0.2
20		1.71659 ± 3E-05	-0.5	700 ± 50	7.9 ± 0.3
21		1.71673 ± 4E-05	0.5	650 ± 80	11.1 ± 0.4

Fig. S10: Color-code map of the circularly resolved μ-PL intensity as a function of positive and negative out-of-plane magnetic field, at 3.6 K, for the WSe$_2$/BGB-16Tb sample. The peaks can be fitted using Eq.(1) in the main text. The fitting parameters for each analyzed peak are shown in the table on the right side. In addition to spectral wandering and linear polarization, the doublets can also be assigned by the similarity in the obtained parameters. The index j indicates if the peak is red or blue-shifting with the increased magnetic field. The parameters for the doublets mentioned above are highlighted.

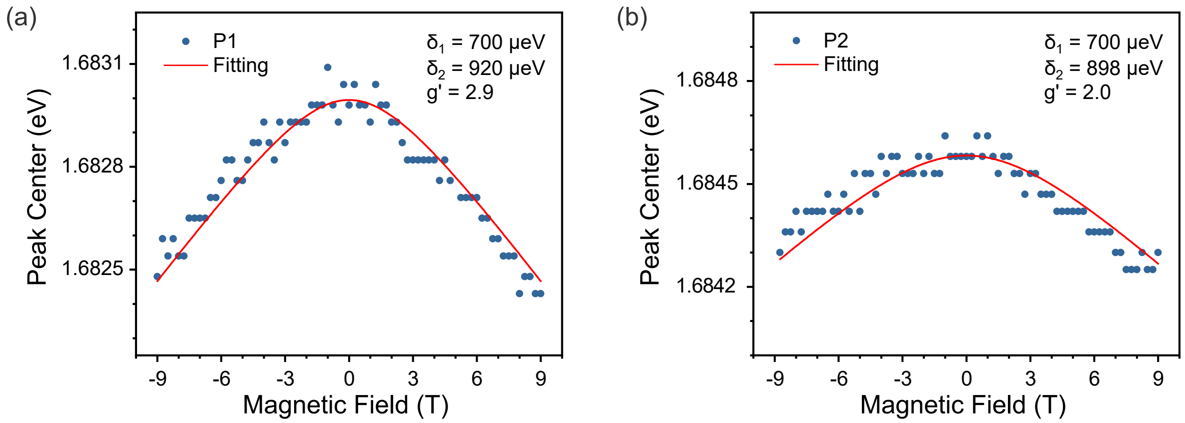

Fig. S11: Extracted peak positions as a function of in-plane magnetic fields for peaks P_1 and P_2, from Figure 3(b) in the main text for the sample WSe$_2$/BGB-16Tb. The data was fitted using Equation (2).

Theoretical Model

The observation of sharp PL peaks with single-photon emitting properties suggests that the WSe_2 layer has both local strain and defect levels. In this case, the model proposed in Ref. [5] demonstrates that the set of four electron-hole pair states illustrated in Figure S12(a) represents an appropriate basis for the ground state exciton states. The combination of strain and confinement in monolayer WSe_2 allows the defect states for spin down $|d\downarrow\rangle$ and spin up electrons $|d\uparrow\rangle$ to participate in transitions with both K and K' holes, since the concept of K/K' valley and their restrict selection rules is ill defined for such a confining system. Density functional Theory(DFT)-based calculations of the excitonic states within this basis, including exchange and direct electron-hole interactions, leads to two pairs of states, separated by an energy δ_2 - the highest energy pair ($|\downarrow K\rangle$ and $|\downarrow K'\rangle$, where the arrows refer to the spin of the electron defect state) is still degenerate, whereas the lowest energy pair, composed by combinations $(1/\sqrt{2})\,(|\uparrow K\rangle \pm |\uparrow K'\rangle)$, exhibit an extra energy split δ_1. The basis states in the lowest energy pair get mixed and show an anti-crossing profile as a perpendicular magnetic field B_z is applied, whereas the higher energy levels remain independent and exhibit a crossing at $B_z = 0$ instead. Also, the low energy pair states are the only bright ones amongst the four eigenstates of the system for any value of B_z.[5] We argue that every anti-crossing pair of states in Figure 2 of the main text refers to a lowest energy pair of hybridized states localized at a different defect – the wide dispersion in defect sizes verified in the AFM results in Figure 1 explains the wide variety of average energies between different defect confined pairs. Therefore, by analyzing the magneto-PL results within the Faraday configuration, one obtains information about the exchange energy δ_1 between the lowest energy pair states, which is found here to be within a range of 580 to 740 μeV.

On the other hand, as an in-plane magnetic field is applied to the sample (Voigt configuration), one experimentally observes that, for each pair of bright exciton states, both states undergo a significant redshift, as seen in Figure 3 of the main text. Such a redshift, which

has not been experimentally observed yet, to the best of our knowledge, brings information about the exchange interaction between the two pairs of states, split by an energy δ_2. A model Hamiltonian that explains the behavior of the lowest energy exciton pairs for both Faraday and Voigt magnetic field configurations in the basis $(|\downarrow K\rangle \; |\downarrow K'\rangle \; |\uparrow K\rangle \; |\uparrow K'\rangle)$ is

$$H = \begin{pmatrix} \delta_2/2 + g\mu_B B_z/2 & 0 & g'\mu_B B_x/2 & 0 \\ 0 & \delta_2/2 - g\mu_B B_z/2 & 0 & g'\mu_B B_x/2 \\ g'\mu_B B_x/2 & 0 & -\delta_2/2 + g\mu_B B_z/2 & \delta_1/2 \\ 0 & g'\mu_B B_x/2 & \delta_1/2 & -\delta_2/2 - g\mu_B B_z/2 \end{pmatrix} \quad (1)$$

where $g(g')$ is the effective Landé g-factor of the exciton for a magnetic field $\vec{B}$ applied in the out-of-(in-)plane direction, and the exchange energy parameters $\delta_{1(2)}$ control the energy separation between exciton states. The in-plane direction is chosen here as the x-direction, without loss of generality of the theoretical results.

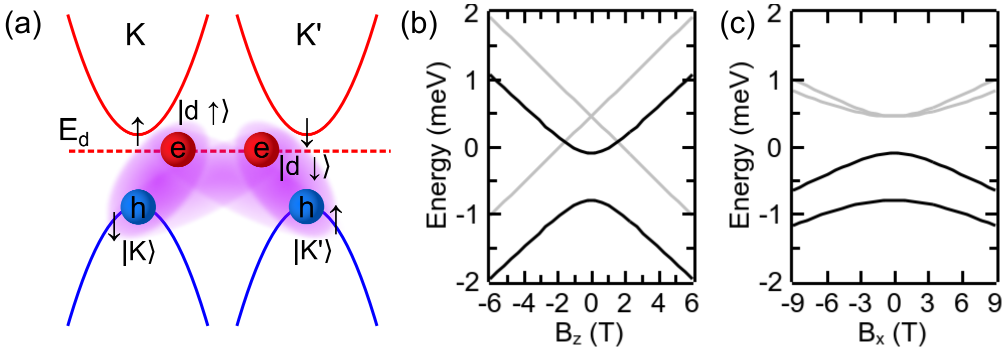

Fig. S12: (a) Sketch of the single-particle states involved in the excitons in the samples investigated here. Defect states for electrons, represented by the horizontal dashed line, mix conduction band states from K and K' valleys, forming $|d\uparrow\rangle$ and $|d\downarrow\rangle$ states for spin up and down electrons, respectively. These states can be bound to both K and K' hole states, as represented by the shaded ellipses in the sketch, forming four types of exciton. Exchange interactions lead to the final eigenstates of the system as a linear combination of these four excitonic types. (b) Exciton energies obtained by diagonalization of the Hamiltonian H in Eq. (1) as a function of an out-of-plane magnetic field B_z, for $B_x = 0$. Black (gray) lines represent optically brigh (dark) excitonic states. (c) The same as in (b), but for a non-zero in-plane magnetic field B_x, and $B_z = 0$.

Assuming a strictly out-of-plane field, i.e. $B_x = 0$, diagonalization of H leads to four eigenenergies: $\delta_2/2 \pm g\mu_B B_z/2$, and $-\delta_2/2 \pm \sqrt{(\delta_1/2)^2 + (g\mu_B B_z/2)^2}$. The former (latter) two states are the dark (bright) exciton states, whose energies are shown as gray (black) curves in Figure S12(b) as a function of the magnetic field intensity B_z. For the example in Figure S12(b), we have assumed $\delta_1 = 0.7$ μeV and $g = 8.5$, which are within the range of experimentally obtained values. We have also assumed $\delta_2 = 1$ meV in this example, although the energies of the bright exciton states under B_z do not depend on this parameter. Notice the results obtained with H are in good agreement with those from DFT calculations of Ref. [5], and the B_z-dependence of the bright states corroborates the experimental results in Figure 2 of the main text, which helps to validate our model.

By employing the same model for $B_z = 0$, aiming at modelling a system under Voigt configuration, diagonalization of H leads to four eigenenergies, $1/2[\delta_1/2 \pm \sqrt{(\delta_2 - \delta_1/2)^2 + (g'\mu_B B_x)^2}]$ and $1/2[-\delta_1/2 \pm \sqrt{(\delta_2 + \delta_1/2)^2 + (g'\mu_B B_x)^2}]$, which are shown in Figure S12(c) as a function of the magnitude of the in-plane field B_x. In this case, the energy of both bright exciton states decrease with the field, just as observed in the experiment. Here, the exchange energy δ_2 plays an important role in the magnetic field dependence of the energy states, which allows us to use the results in the Voigt configuration to estimate this parameter from experiments.

In the absence of strain and defects, the angular momentum of K/K'-valley conduction and valence band states in monolayer TMDs is locked in the out-of-plane direction by orbital symmetry and spin-orbit coupling. As a consequence, applying an in-plane magnetic field, in principle, should not lead to significant energy shifts in the exciton states, as experimentaly verified e.g. in Ref. [6] for a pristine WSe_2 monolayer. Indeed, notice the Hamiltonian H bears resemblance with the one proposed in Ref. [6] in the context of brightening dark exciton states in monolayer WSe_2 via in-plane fields. However, the role played by the energy parameter δ_2 in H here is played by the electron spin-orbit splitting energy Δ_{SO} in their model. As such, in their case, in-plane fields are still expected to lead to negligible energy shifts in monolayer WSe_2, since $\Delta_{SO} \gg g'\mu_B B_x$ and $\Delta_{SO} \gg \delta_1$ in this material, so that

the exciton eigenenergies are not significantly affected by B_x in the range of fields studied either here or in Ref. [6]. Conversely, Δ_{SO} is only a few meV in $MoSe_2$, which explains the experimentally measurable[7] energy shifts as a function of B_x within the same model. In this case, the Zeeman shift in the Voigt configuration is modelled with the usual $g = 2$ for conduction band electrons, thus reinforcing that the energy shifts originate in an in-plane spin reorientation of the electron state due to the applied in-plane field, as in the model proposed here.

In both WSe_2/BGB-0Tb and WSe_2/BGB-16Tb samples, the defect states behave as SPE with $g \approx 8.5$, but only the latter is affected by an in-plane field. This is a consequence of the stronger strain profile in the latter, which relaxes the condition that locks the electron angular momentum direction, whereas the behavior of the former is still reminiscent of the WSe_2 monolayers in previous experimental works. Thus, even though the model hamiltonian H can be used for both samples, it is expected to be more accurate for the exciton picture present in the WSe_2/BGB-16Tb case, where strain is stronger.

In our sample, in the Voigt configuration, we assume that the angular momentum involved in the Zeeman effect is dominated by spin and orbital contributions in the defect state that are not locked in the out-of-plane direction in the strongly strained (WSe_2/BGB-16Tb) sample, so that the electron involved in the excitonic doublets must have an effective g-factor of the order of $g' \approx 2 - 4$. Energy redshifts due to the in-plane field are observed within 43-98 μeV/T in Figure 3 of the main text. Taking $\delta_2 = 0.9$ meV and $g' = 3$, theoretical results in Figure S12(c) lead to 62 μeV/T, in good agreement with the experimental range. This sets the order of magnitude of δ_2, which also corroborates with the energy splitting obtained via DFT in Ref. [5]. Therefore the redshifts observed in our experimental data supports our interpretation of δ_2 as an exchange energy in a system that combines strain and defect states to form excitons, which is a physically different environment as compared to the previous experimental works involving pristine WSe_2 monolayers, where such redshifts have not been observed.

References

1. Tonndorf, P.; Schmidt, R.; Schneider, R.; Kern, J.; Buscema, M.; Steele, G. A.; Castellanos-Gomez, A.; van der Zant, H. S. J.; de Vasconcellos, S. M.; Bratschitsch, R. Single-photon emission from localized excitons in an atomically thin semiconductor. *Optica, Vol. 2, Issue 4, pp. 347-352* **2015**, *2*, 347–352.

2. Koperski, M.; Nogajewski, K.; Arora, A.; Cherkez, V.; Mallet, P.; Veuillen, J. Y.; Marcus, J.; Kossacki, P.; Potemski, M. Single photon emitters in exfoliated WSe2 structures. *Nature Nanotechnology 2015 10:6* **2015**, *10*, 503–506.

3. Michaelis de Vasconcellos, S.; Wigger, D.; Wurstbauer, U.; Holleitner, A. W.; Bratschitsch, R.; Kuhn, T. Single-Photon Emitters in Layered Van der Waals Materials. *physica status solidi (b)* **2022**, *259*, 2100566.

4. Dang, J.; Sun, S.; Xie, X.; Yu, Y.; Peng, K.; Qian, C.; Wu, S.; Song, F.; Yang, J.; Xiao, S.; Yang, L.; Wang, Y.; Rafiq, M. A.; Wang, C.; Xu, X. Identifying defect-related quantum emitters in monolayer WSe2. *npj 2D Materials and Applications* **2020**, *4*, 1–7.

5. Linhart, L.; Paur, M.; Smejkal, V.; Burgdörfer, J.; Mueller, T.; Libisch, F. Localized Intervalley Defect Excitons as Single-Photon Emitters in WSe2. *Physical Review Letters* **2019**, *123*, 146401.

6. Molas, M. R.; Slobodeniuk, A. O.; Kazimierczuk, T.; Nogajewski, K.; Bartos, M.; Kapuściński, P.; Oreszczuk, K.; Watanabe, K.; Taniguchi, T.; Faugeras, C.; Kossacki, P.; Basko, D. M.; Potemski, M. Probing and Manipulating Valley Coherence of Dark Excitons in Monolayer WSe2. *Physical Review Letters* **2019**, *123*, 096803.

7. Lu, Z.; Rhodes, D.; Li, Z.; Tuan, D. V.; Jiang, Y.; Ludwig, J.; Jiang, Z.; Lian, Z.; Shi, S.-F.; Hone, J.; Dery, H.; Smirnov, D. Magnetic field mixing and splitting of bright and dark excitons in monolayer MoSe2. *2D Materials* **2019**, *7*, 015017.
